# Recognising and managing diabetic retinopathy

**Published:** 2011-09

**Authors:** Anthony Hall

**Affiliations:** Former Head of Department of Ophthalmology, Kilimanjaro Christian Medical Centre, Tanzania. Email: abhall@kcco.net

**Figure:**
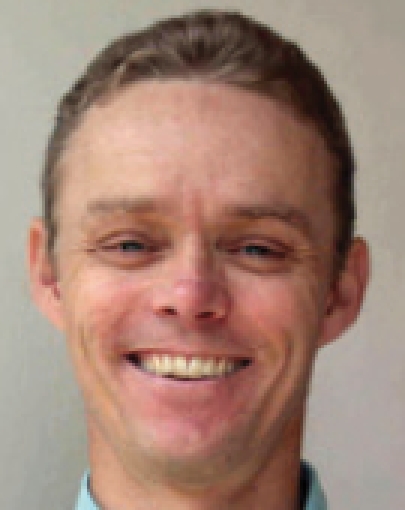

**Key learning points**

- Detecting and diagnosing diabetic retinopathy is not complicated. There are clinical signs which can be seen with an ophthalmoscope or a slit lamp and 90- or 78-dioptre lens.
- Diabetic retinopathy is treatable. Treatment usually maintains vision, but does not restore vision that has already been lost.
- In diabetic maculopathy, laser or anti-VEGF injections are both proven to work. Intravitreal steroid is ineffective in most patients.
- Laser treatment should use small spots and just enough power to produce a visible reaction.
- Proliferative retinopathy is best treated with pan-retinal laser. The commonest error is undertreatment, and laser should be applied until there is regression of the new vessels or there is no room for further treatment.
- Vitrectomy is useful for vitreous haemorrhage and late complications of proliferative retinopathy. Pre-treatment with bevacizumab reduces the risk of surgical complications.

**Figure 1:**
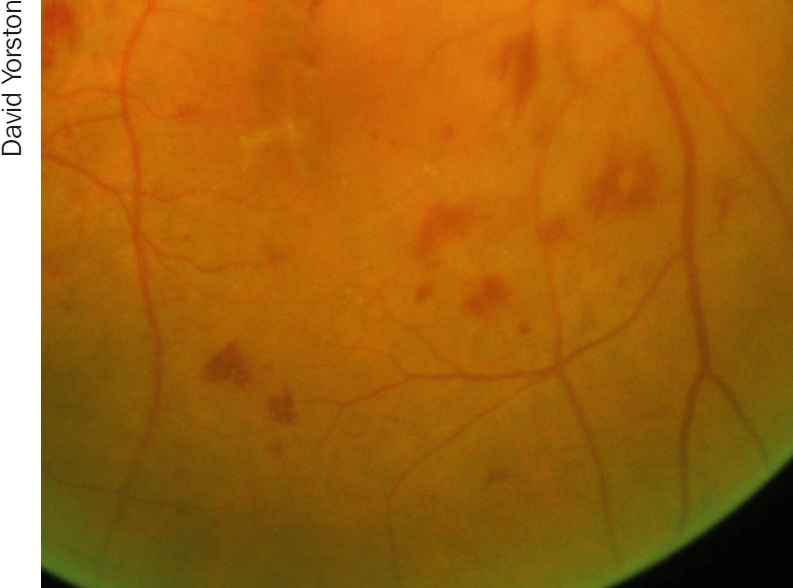
Haemorrhages (larger, uneven red ‘blots’) and microaneurysms (small, round ‘dots’)

**Figure 2:**
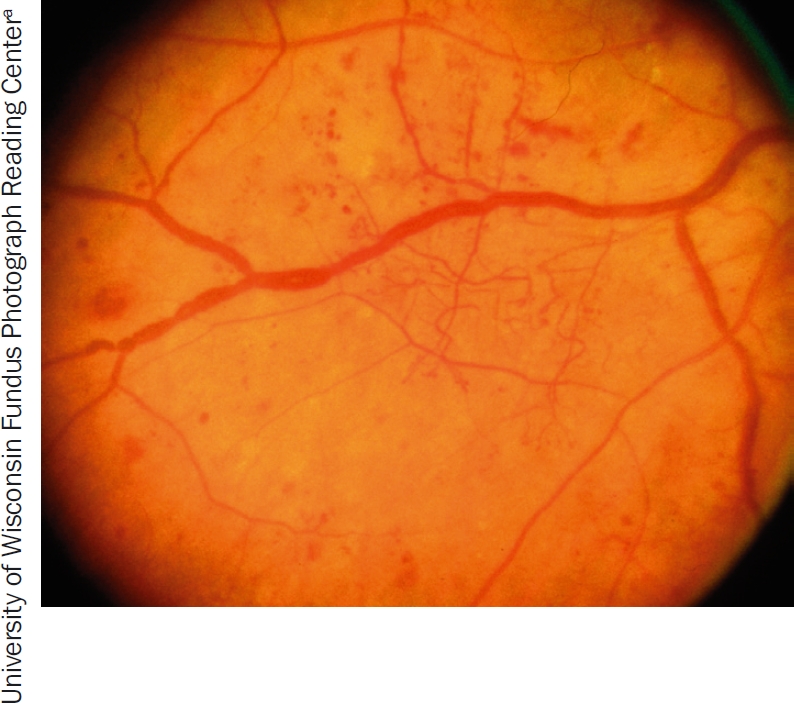
Venous beading, i.e., irregular calibre (‘thickness’) of the veins

**Figure 3:**
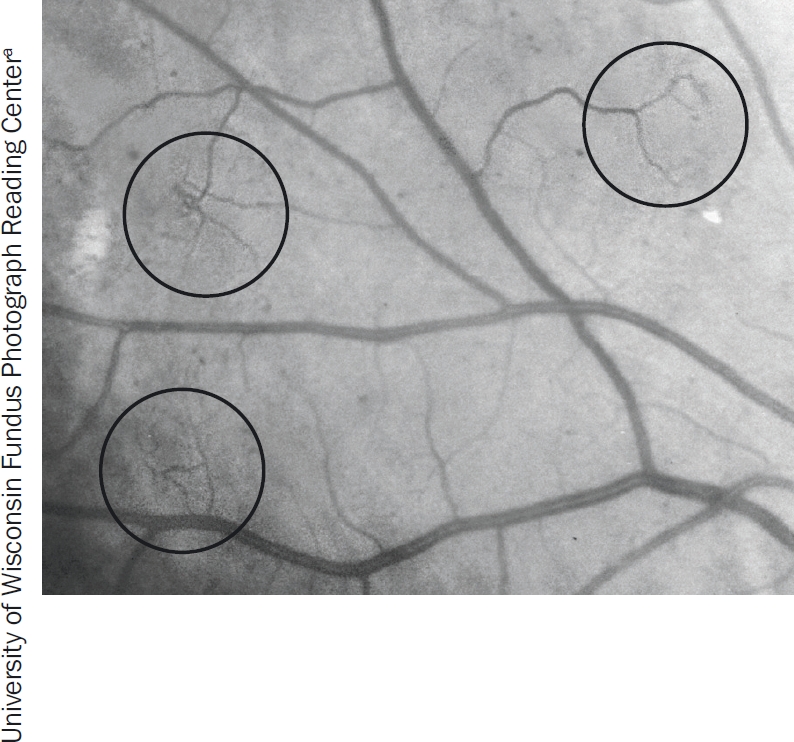
Intraretinal microvascular abnormalities (IRMA). The circles show the odd, twisted shape of IRMA

## Recognising DR

The management of diabetic retinopathy (DR) depends on accurately recognising or classifying the different types of DR and knowing what treatment to give the patient.

DR has clinical signs which can be seen with an ophthalmoscope or with a slit lamp and a 90- or 78-dioptre lens. The advantage of the slit lamp is that it allows you to visualise the retina with both eyes. This stereoscopic vision provides a sense of depth which aids diagnosis, particularly of macular oedema. Other aids to DR diagnosis are fundus photography, fluorescein angiography, and optical coherence tomography (see box on page 7).

### 1 Non-proliferative DR

The clinical signs of **non-proliferative DR** are:

- haemorrhages (Figure 1)
- microaneurysms (Figure 1)
- venous beading (Figure 2)
- intraretinal microvascular abnormalities (IRMA) (Figure 3)

### 2 Proliferative DR

**Figure 4:**
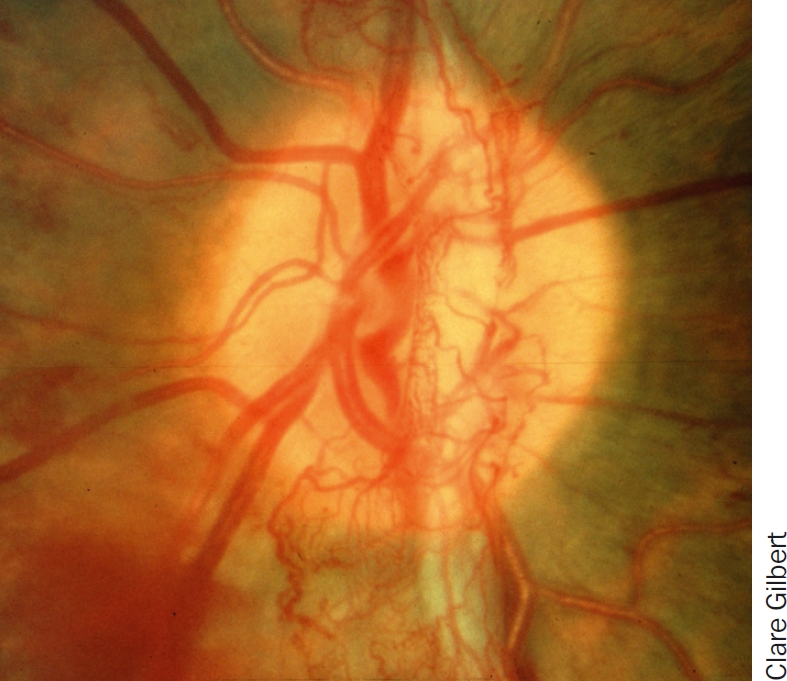
New vessels, the key characteristic of proliferative diabetic retinopathy

Proliferative DR can exhibit all the same clinical signs as non-proliferative DR. However, the key characteristic of proliferative DR is new vessels growing onto the posterior vitreous surface from the retina or optic disc (Figure 4).

**Figure 5:**
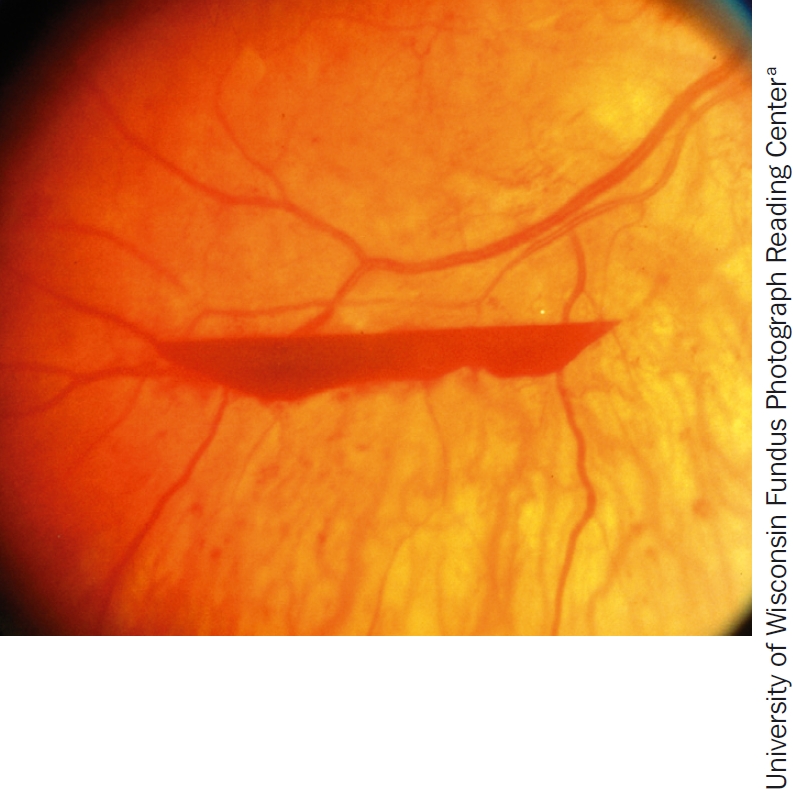
Pre-retinal haemorrhage, one of the signs of proliferative diabetic retinopathy

The new vessels damage sight by bleeding (Figure 5) or forming sheets of fibrovascular membranes that may cause traction retinal detachments. Traction retinal detachment occurs when the fibrovascular tissue contracts and pulls the retina away from the underlying choroid. If this affects the macula, the central vision will be lost.

The clinical signs of **proliferative DR** include:

- new vessels growing onto the posterior vitreous surface from the retina or optic disc (Figure 4)
- vitreous and/or pre-retinal haemorrhages (Figure 5)
- fibrosis
- traction retinal detachment.

### 3 Diabetic maculopathy

Diabetic maculopathy occurs when DR affects the central part of the retina. Blood vessels leak, leading to diabetic **macular oedema** (swelling of the retina).

**Figure 6:**
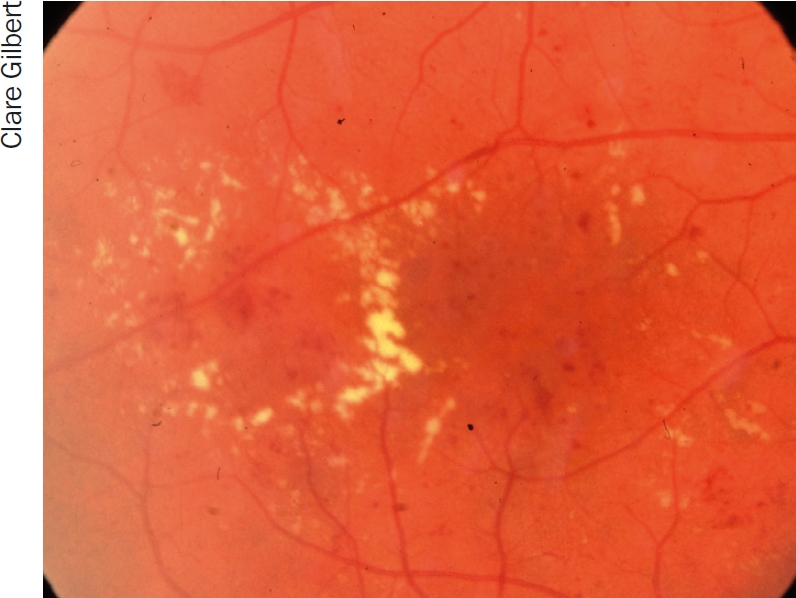
Exudates. This is an example of circinate exudates, which are circular in appearance

The early treatment of diabetic retinopathy study (ETDRS) defined clinically significant macular oedema (CSMO)^1^ as the stage at which the eye needs to be treated in order to prevent loss of vision. The definition depends on recognising the following:

- retinal thickening and exudates (Figure 6) at or within 500 microns of the fovea (within one third of a disc diameter).
- larger zones of retinal thickening (one disc diameter or more), if within one disc diameter of the fovea.

Retinal thickening can only be observed stereoscopically. So, for practical clinical purposes, look for other easily visible markers for macular oedema such as exudates within a disk diameter of the fovea.

The blood vessels in the central part of the retina may also become blocked (capillary closure), leading to **macular ischaemia**. Macular ischaemia occurs when there is insufficient blood supply to the macula. This will impair the normal functioning of the retina, leading to reduced vision.

There is no effective treatment for macular ischaemia,^1^ but it is important to recognise it so that you don't waste the patient's time and money with ineffective laser or anti-vascular endothelial growth factor (anti-VEGF) treatment.

**Figure 7:**
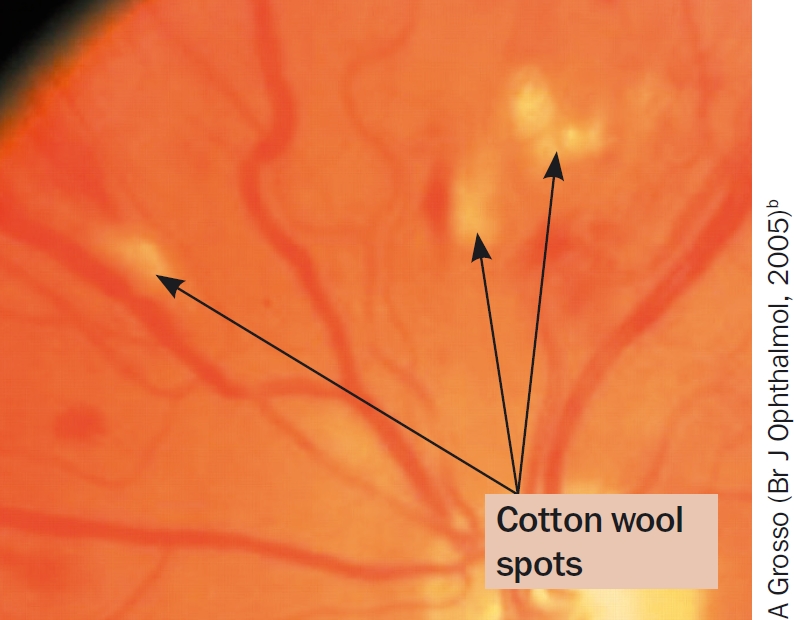
Cotton wool spots

Although macular ischaemia can only be diagnosed conclusively by fluorescein angiography (see box on page 7), you should suspect it if the following conditions are met:

- reduced visual acuity
- evidence of retinal ischaemia, e.g. cotton wool spots (Figure 7) or blot haemorrhages
- no macular oedema at the fovea
- no other cause for reduced vision (e.g. cataract, refractive error).

**FROM THE FIELD: How I look for diabetic retinopathy: a vision technician's experience**

**Figure:**
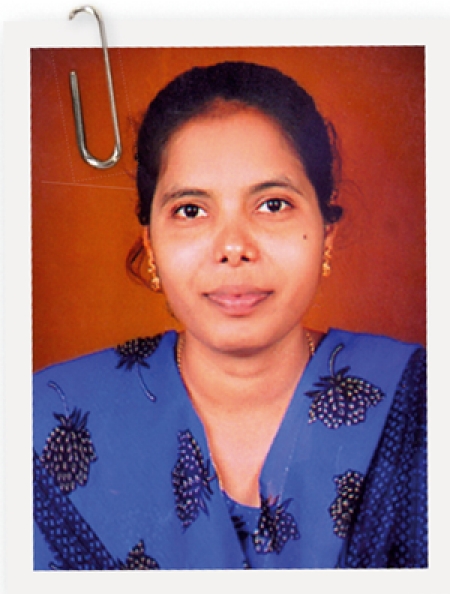

“I am Lalitha Y, and I have been working as a vision technician at LV Prasad Eye Institute since 2008. I hail from a remote rural village in Prakasham District and I joined the vision technician programme at LVPEI in 2007. During this programme, we were trained to look for diabetic retinopathy (DR) by direct ophthalmoscope.

“Whenever someone with diabetes visits my vision centre, I will initially take a detailed history regarding the duration of diabetes, their blood sugar control, medication (drugs/insulin), diet, physical activity, smoking, alcohol intake, family history of diabetes, and other systemic diseases like hypertension, diabetic nephropathy, neuropathy, and so on. I also record any history of blurred vision for distance or near vision, flashes, floaters in the field of view, and any fluctuations in vision.

“After checking the patient's visual acuity, I check for any extra-ocular muscle imbalance by checking eye movements in all directions. During a slit lamp examination (before dilation) I look mainly for neovascularisation of the iris and record intraocular pressure.

“After dilation, I will then examine the posterior segment by direct ophthalmoscope. If the media are clear, I will check for signs of DR, such as haemorrhages or exudates. If the media are not clear or if the patient has signs of DR, I will refer the patient to a secondary centre ophthalmologist for dilated fundus examination, which will give them the information they need to manage the patient's DR. Recently, I was trained to take fundus photographs. This helps me to identify patients with DR, who I would then send to a secondary centre for further management.

**‘I will initially take a detailed patient history’**

“I will then talk to the patient and explain the role of good blood sugar control.”

**Editor's note**: In patients with high blood pressure, good blood pressure control will reduce the likelihood that a patient's DR will get worse (see article on page 4). Eye care workers would do well to check their patients' blood pressure and advise those with high blood pressure on the importance of control, referring them to a physician if they needed help.

## Treating proliferative diabetic retinopathy

The two main treatment options for proliferative DR are pan-retinal laser photocoagulation and diabetic vitrectomy.

### Pan-retinal photocoagulation (PRP)

Pan-retinal photocoagulation (PRP), or scatter laser, is the main form of treatment for proliferative diabetic retinopathy.

The aim of the laser is to induce regression of new blood vessels (that is, to make them stop growing and shrink). It must be given early enough and cover enough retina to induce regression of the vessels that cause the complications of vitreous haemorrhage and tractional detachment of the retina.

A 50% reduction in severe visual loss after PRP was reported by the Diabetic Retinopathy Study for patients with new vessels on the optic disc.^1^

### Diabetic vitrectomy

Vitrectomy is indicated in proliferative diabetic retinopathy in the following conditions:

- non-clearing vitreous haemorrhage
- pre-retinal (or sub-hyaloid) haemorrhage
- tractional retinal detachment threatening, or involving, the macula
- combined rhegmatogenous/tractional detachment
- progressive severe fibrovascular proliferation in spite of adequate PRP

Currently, vitrectomy for diabetic macular oedema is reserved for the few patients who have vitreous traction on the macula.^2^

The technique is an important part of the treatment of proliferative diabetic retinopathy and leads to improvement or stabilisation of vision in 90% of patients.^1,2^ Vitreous and blood are cut and aspirated and membranes causing tractional detachment of the retina are removed. This may be done by segmenting the membranes or by delamination, i.e. removing the whole of the posterior hyaloid and associated fibrovascular membranes by cutting them off the surface of the retina.

In countries without screening, many people present with long-standing tractional retinal detachments of the macula. The result of diabetic vitrectomy in these eyes is not so good. In a resource-poor environment, those with a better prognosis should be prioritised.

It is worth pre-treating patients with **intravitreal bevacizumab** prior to vitrectomy.^3^ A Cochrane review of six randomised controlled trials found that pre-treatment with 1.25 mg of intravitreal bevacizumab resulted in shorter operations with less endodiathermy and intra-operative bleeding. Post-operative reabsorption of blood was significantly shorter. Final best-corrected visual acuity was significantly better.

The effect of intravitreal bevacizumab on neovascularisation is rapid. The first effects can be seen in 24 hours. The optimum time for a preoperative injection would seem to be 5-7 days before the operation.

In a proportion of patients, intravitreal bevacizumab preoperatively may lead to clearing of the vitreous haemorrhage, thus avoiding surgery.

## Treating diabetic maculopathy

Diabetic maculopathy is a major cause of vision loss amongst patients with diabetes. Treatment includes steroids, anti-vascular endothelial growth factor (anti-VEGF), and laser.

### Steroid treatments

In the Diabetic Retinopathy Clinical Research Network trial, intravitreal injections of the steroid triamcinolone acetonide was compared with standard laser treatment. Although there were short-term improvements in visual acuity with intravitreal triamcinolone acetonide (IVTA), this improvement was not sustained. Laser was more effective and had fewer side effects than IVTA. The side effects of IVTA included cataract formation and raised intraocular pressure. Recently, the same group found that there was one exception. In pseudo-phakic eyes, IVTA and prompt laser seemed more effective than laser alone.^4^

**Investigating DR: photos, fluorescein angiography and OCT**

**Figure:**
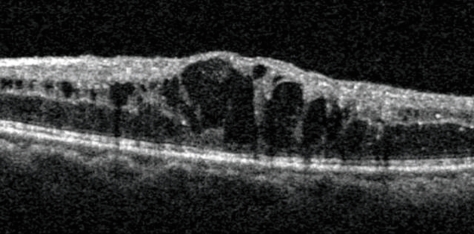
An optical coherence tomography (OCT) image of cystoid macular oedema

Diabetic retinopathy (DR) can be diagnosed by clinical examination alone if you are good at examining the retina with a slit lamp microscope. Using the slit lamp, you will be able to detect haemorrhages, new vessels, exudates, and retinal thickening due to oedema. If the diagnosis can be made clinically, are investigations ever necessary?

**Photos** are probably the most useful investigation. The cost of fundus cameras is still high, but they are becoming more affordable and the quality of pictures is improving all the time. They are also easy to use. The most valuable use of photography is in patients with diabetic maculopathy or new vessels who have laser treatment. Often, the laser leads to a complete cure and the exudates and new vessels disappear. However, sometimes they do not completely resolve. If you only examine the patient occasionally, it is difficult to remember exactly what the retina looked like before you treated it. When you can still see retinopathy months after laser treatment, you may be unsure if it is better, worse, or much the same. If you have photos to refer to, you can be certain of what has changed. Of course, photos are also very useful for detecting DR and counselling patients.

**Fluorescein angiography** is a technique for examining the fine detail of the retinal circulation. It will show the leaks that cause exudative maculopathy and the areas of blocked capillaries that cause ischaemic maculopathy and proliferative retinopathy. However, injections of fluorescein carry a small risk (about 1:20,000) of a severe allergic response, which can be fatal. They should not be given unless there are facilities for resuscitation.

**‘If you have photos to refer to, you can be certain of what has changed’**

**Optical coherence tomography (OCT)** is a relatively new technique that uses lasers to scan the retina and build up a very detailed three-dimensional image. This will not only detect any oedema or swelling of the retina, but also measure it and draw a map that shows the areas where the swelling is greatest. It is fast, safe, and does not require any injections. Unfortunately, the machines cost about £50,000! In high-income countries, OCT and photos, in combination, are the usual means of documenting and investigating DR. As cameras and OCT machines become more affordable, they will also become more widely used in low- and middle income countries.

### Anti-vascular endothelial growth factor (anti-VEGF) treatment

VEGF levels are increased in the vitreous and retina in patients with diabetic retinopathy. The most recent anti-VEGF drugs to be evaluated in the treatment of diabetic maculopathy are ranibizumab^2^ (Lucentis) and bevacizumab^5^ (Avastin). These trials showed a benefit with intravitreal ranibizumab and bevacizumab in patients with foveal thickening. However, intravitreal ranibizumab injections cost around US $1,200 each and the patients in this study received eight or nine injections in the first year (a cost of around US $10,000 per patient per year.) Intravitreal bevacizumab is much cheaper. We are able to offer patients an intravitreal bevacizumab injection for as little as US $25.

In practice, laser should remain the cornerstone of treating clinically significant macular oedema and the use of intravitreal injections should be tailored to the needs of individual patients.

### Laser

The Early Treatment of Diabetic Retinopathy study compared macular laser with observation. There was a 50% reduction in moderate visual loss in the group that received laser (from 24% to 12%).

The recommended protocol is as follows:

- Treat circinate exudates (Figure 6) with focal laser, blanching the retina in the centre of the exudate. It is not necessary to target individual microaneurysms.
- Diffuse macular oedema is treated by a grid laser in the area of thickening. Burns should be one burn width apart, using a spot size of 75 to 125 microns, duration 20-50 milliseconds. Do not use the repeat mode.
- Start with a low power setting, around 150 milliwatt, and increase the power until the desired endpoint is reached. Aim to produce a grey to cream change in colour. White means the laser is too hot and the power should be reduced. Take care not to encroach on the foveal avascular zone. It is wise to avoid treating perifoveal microaneurysms as this is likely to increase perifoveal capillary dropout (consider intravitreal bevacizumab instead). The chorioretinal atrophy caused by burns, especially intense burns, within 300 to 500 microns of the fovea can years later extend into the fovea and cause vision loss, particularly in myopes.
- In patients with established foveal thickening or who are not responding to laser, consider intravitreal bevacizumab. In pseudophakic eyes, consider IVTA but watch the intraocular pressure closely.

**Managing diabetic retinopathy in Africa**

**Case study**

In our clinic, a typical patient, Mrs X, was first seen with a visual acuity of 6/9, a few macular exudates, and proliferative disease. The treatment plan followed the textbook recommendation of doing focal laser for the maculopathy first. The patient then missed two appointments and pan-retinal photocoagulation (PRP) was delayed by about two months. When PRP was finally given, the intention was to give it in the recommended multiple sessions. However, due to further missed appointments, the interval between laser sessions was over a month. This allowed fibrovascular proliferation to continue. It was six months from the time of presentation before laser was completed. By then, tractional retinal detachment involving the macula had developed and vitrectomy was required. Mrs X's final visual acuity was counting fingers at three metres.

What are the lessons to be learned from this? How can we do better?

We audited a number of patients who had ultimately needed vitrectomy for advanced proliferative disease to find out how we could improve, and arrived at the following recommendations for laser in countries where patients may not come for regular appointments.

**Recommendations**

**Figure 8:**
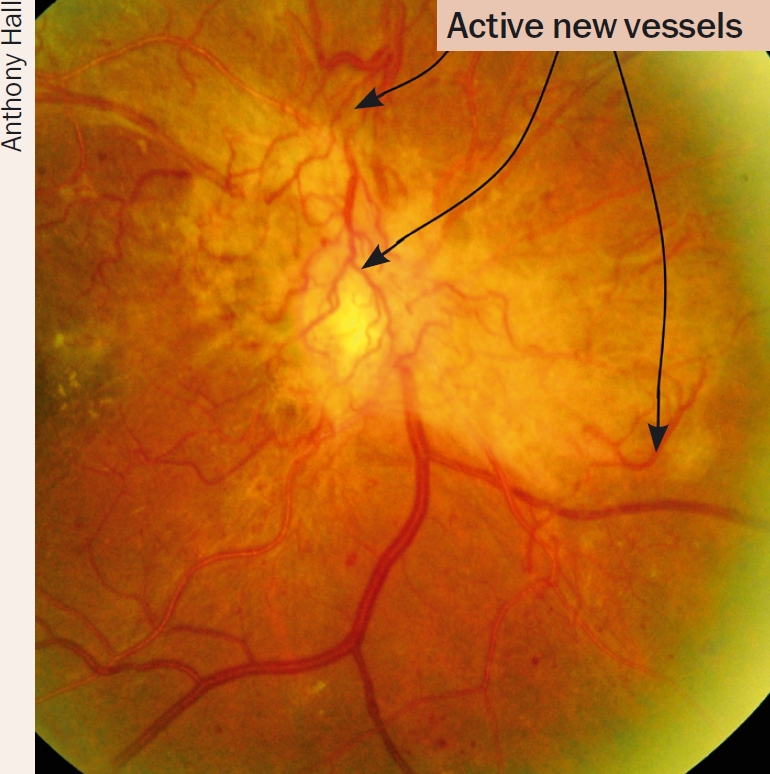
Eye before treatment. The arrows at the top point out active new vessels

**Figure 9:**
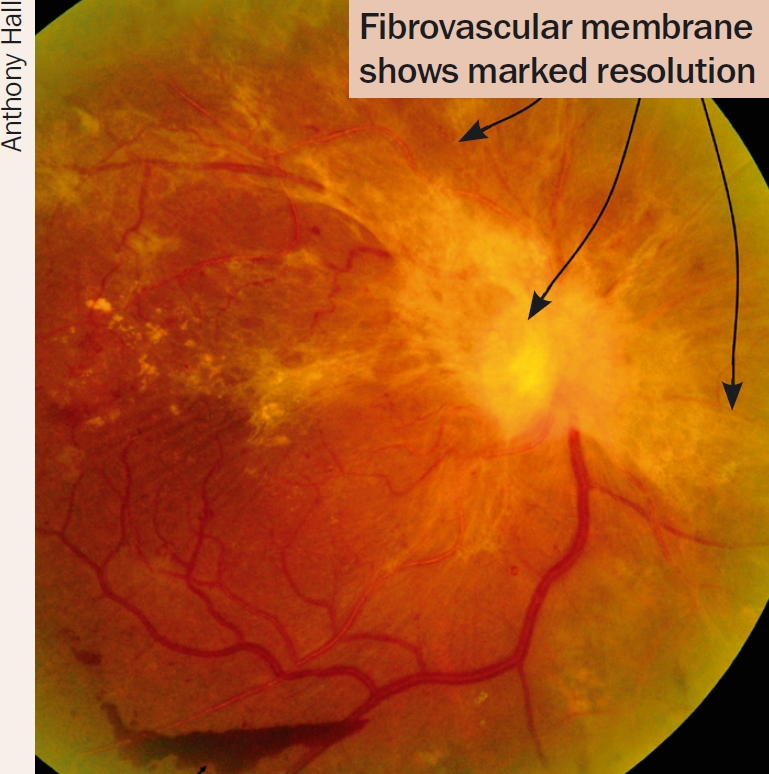
Ten days after PRP. Note regression of new vessels

**Figure 10:**
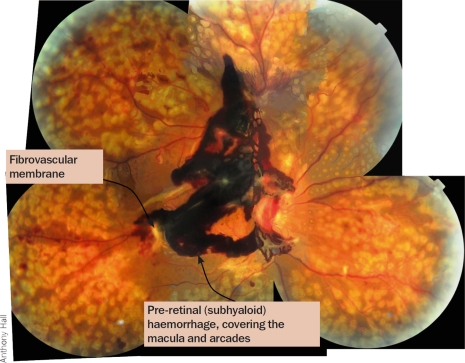
Pan-retinal photocoagulation

1. **Warn all diabetes patients to come if they experience floaters or blur**, as these symptoms suggest a vitreous haemorrhage.
2. **Give PRP to anyone who has vitreous or sub-hyaloid blood** (Figure 5) even if there are no visible new vessels. Treat any size area of definite neovascularisation, on the disc or elsewhere. Treat any eye that has evidence of fibrosis, as this is evidence of proliferative disease.
3. **Treat patients faster**. Regression of new vessels should be seen after a week or two. (Figures 8 and 9). If patients come from far away, consider admitting them to complete the laser before they are discharged. Attempt to complete the laser in one week, instead of several weeks. Treat the inferior retina first as new blood falls down and blocks the view inferiorly.
4. **Make the most of each session you have**. It is worth treating some patients in one session. This is particularly important if there are large neovascular (NV) formations which have an increased risk of bleeding, or if the patient is unlikely to return. Remember to avoid application of intense burns which are unnecessary to induce regression. A pan-retinal pattern of excessively intense burns can lead to choroidal effusion and angle-closure glaucoma with blindness. Treat one burn width apart, as shown in Figure 10. Oedema surrounding the burns makes them look more confluent than they are.
5. **Repeat treatment**. All neovascularisation should regress in two to four weeks. If it has not regressed, treat again. If bleeding occurs after laser, re-treat until NV formations have gone or maximal treatment has been given. Consider treating inside the arcades, particularly temporally.

Populations in low- and middle-income countries face a huge burden of blinding DR. Urgent advocacy is needed for governments to initiate programmes to address this. In the interim, every residency programme must provide training in the skills needed to manage DR, including interpreting investigations and delivering laser and other treatments. Refresher courses can be arranged for those not adequately trained or who have been without the necessary equipment for some time. We must also advocate for lasers and other necessary equipment wherever there is a trained ophthalmologist.
